# Video evidence that parenting methods predict which infants develop long night-time sleep periods by three months of age

**DOI:** 10.1017/S1463423616000451

**Published:** 2016-12-28

**Authors:** Ian St James-Roberts, Marion Roberts, Kimberly Hovish, Charlie Owen

**Affiliations:** Thomas Coram Research Unit, UCL Institute of Education, University College London, London, UK

**Keywords:** infant crying, infant sleeping, parenting

## Abstract

**Aim:**

To examine two hypotheses about the longitudinal relationship between night-time parenting behaviours in the first few postnatal weeks and infant night-time sleep-waking at five weeks, three months and six months of age in normal London home environments.

**Background:**

Most western infants develop long night-time sleep periods by four months of age. However, around 20–30% of infants in many countries continue to sleep for short periods and cry out on waking in the night: the most common type of infant sleep behaviour problem. Preventive interventions may help families and improve services. There is evidence that ‘limit-setting’ parenting, which is common in western cultures, supports the development of settled infant night-time behaviour. However, this evidence has been challenged. The present study measures three components of limit-setting parenting (response delay, feeding interval, settling method), examines their stability, and assesses the predictive relationship between each of them and infant sleep-waking behaviours.

**Methods:**

Longitudinal observations comparing a General-Community (*n*=101) group and subgroups with a Bed-Sharing (*n*=19) group on infra-red video, diary and questionnaire measures of parenting behaviours and infant feeding and sleep-waking at night.

**Findings:**

Bed-Sharing parenting was highly infant-cued and stable. General-Community parenting involved more limit-setting, but was less stable, than Bed-Sharing parenting. One element of General-Community parenting – consistently introducing a short interval before feeding – was associated with the development of longer infant night-time feed intervals and longer day-time feeds at five weeks, compared with other General-Community and Bed-Sharing infants. Twice as many General-Community infants whose parents introduced these short intervals before feeding in the early weeks slept for long night-time periods at three months of age on both video and parent-report measures, compared with other General-Community and Bed-Sharing infants. The findings’ implications for our understanding of infant sleep-waking development, parenting programmes, and for practice and research, are discussed.

Waking and crying out at night is reported in around 20–30% of infants in many countries (Sadeh and Sivan, 2009; Mindell *et al*., 2010), making this the earliest and most common type of infant sleep behaviour problem. Interventions which prevent this problem may help many families and improve services.

Building on evidence that most western infants develop long sleep periods at night by four months of age (Moore and Ucko, 1957; Anders *et al*., 1992; Henderson *et al*., 2010), four randomised-controlled trials (RCTs) showed that ‘limit-setting’ parenting increased the number of infants with long night-time sleep periods, and reduced night-time ‘signalling’ (crying out), during this key stage for sleep-waking development (Wolfson *et al*., 1992; Pinilla and Birch, 1993; St James-Roberts *et al*., 2001; Symon *et al*., 2005). ‘Limit-setting’ parenting is common in western societies, employing routines and delayed responding to encourage infants to develop autonomous settling (Jenni and O’Connor, 2005; St James-Roberts *et al*., 2006). In contrast, ‘infant-cued’ parenting, which includes high proximity, rapid responses and, in some cases, bed-sharing, is associated with persistent infant signalling at night (St James-Roberts *et al*., 2006; Sadeh *et al*., 2010; Hysing *et al*., 2014). Importantly, limit-setting parenting increased breast-fed infants’ night-time sleep lengths by four months without affecting weight gain (Pinilla and Birch, 1993; St James-Roberts *et al*., 2001), indicating compatibility with breast-feeding.

Given this evidence, it is puzzling that two attempts to apply these findings to community health services did not increase the numbers of infants with long night-time sleep periods (Stremler *et al*., 2013; Hiscock *et al*., 2014). This may be because limit-setting parenting was common in the communities involved so that intervention and control groups were too similar, as Stremler *et al*. (2013) argue. However, a recent report has concluded that limit-setting parenting does not support sleep-waking development and risks increasing infant distress (Douglas and Hill, 2013).

Analyses in a linked report examine whether limit-setting parenting is associated with infant distress at night (St James-Roberts *et al*., 2016). In summary, infants in the limit-setting group had around 30 min more distress per night at two weeks (2W), reducing to 12–13 min per night by three months (3M) of age, compared with Bed-Sharing infants. However, excluding Bed-Sharing cases, differences in infant distress between General-Community subgroups adopting limit-setting versus infant-cued parenting were not large or statistically significant at any age.

The present report addresses the review’s other query by examining longitudinal relationships between limit-setting parenting and infant night-time sleeping in the first 3M of age in the same infants and parents. As well as parental reports, we used infra-red video recording methods developed by Anders *et al*. and proven valid for observation of night-time behaviours at home (Anders and Keener, 1985; Goodlin-Jones *et al*., 2001; Sitnick *et al*., 2008). Analyses were guided by two hypotheses as to how limit-setting parenting might encourage the development of infant sleep-waking at night:

(1) Our primary hypothesis was that delayed parenting response at prior ages would reduce infant night-time signalling at 3M and 6M of age. Derived from learning theory and sleep problem treatment studies, this hypothesis posits that immediate parental response maintains infant night-time signalling until later ages, while delayed response extinguishes it. Because some parents vary their care strategies during the postnatal period (Goodlin-Jones *et al*., 2001), we assessed parenting stability, versus instability, in predicting infant sleep-waking.
(2) Our secondary hypothesis was that implementing an interval between infant waking and feeding (rather than feeding immediately) would predict prolonged infant sleep periods, and reduced signalling, at night. This ‘feeding interval’ hypothesis was based on Wright’s (1993) observation that breast-fed infants increase morning feeds to be larger than other feeds by two months of age, presumably in response to their night-time fast. Further, it reflected limit-setting guidelines that parents should introduce short intervals between infant waking and feeding, for example for nappy (diaper) changes, to break the association (Pinilla and Birch, 1993; St James-Roberts *et al*., 2001). We hypothesised that infants whose parents implemented an interval between infant waking and feeding would develop longer night-time inter-feed intervals and longer day-time feeds at five weeks (5W), leading to longer night-time sleep periods at 3M and 6M of age.

## Methods

The study received Riverside Medical Research Ethics Committee approval (REC 09/H0706/11).

### Participants

Two groups of mothers and breast-feeding singleton infants living within the M25 motorway around London, UK were recruited. The ‘General-Community’ group (*n*=101) was enrolled in postnatal wards of a general community maternity hospital. We excluded multiple births, infants with birth weight <2500 g, admitted to special care, who medical staff considered unwell, and mothers with limited English. Otherwise, mothers were approached consecutively, introduced to the study, and asked to allow a telephone call to explain the research fully after returning home. Mothers who gave written informed consent completed the newborn Infant Sleep and Feeding Arrangements Questionnaire (ISFAQ), described below, when infants were <48 h old.

For comparison, we recruited a group of mothers who planned to adopt highly infant-cued parenting. Prior studies found that mothers who intend to bed-share with their babies are likely to adopt infant-cued parenting behaviours in general (St James-Roberts *et al*., 2006; Hysing *et al*., 2014). Mothers included in this study’s planned Bed-Sharing group met the recruitment criteria of the General-Community group but intended to bed-share with their babies most of the night (defined as ⩾90% of the night, ⩾5 nights/week based on the mothers’ ISFAQ answers: see Table 1). Only one mother approached in the maternity hospital met these criteria; most (18 of 19) were recruited during pregnancy via parenting networks. They gave written informed consent and completed the ISFAQ before or within 48 h of their baby’s birth. This group’s size was small despite 18 months of recruitment, possibly because this coincided with medical guidance that bed-sharing is unsafe. Table 1 in St James-Roberts *et al*. (2016) provides detailed recruitment, participant demographic and missing data for General-Community and Bed-Sharing groups.

**Table 1:** The Infant Sleep and Feeding Arrangements Questionnaire (ISFAQ) items

Items^a^	Possible answers
1. How are you planning to feed (currently feeding) your baby?	Breast only Breast+expressed breast milk Breast+formula milk Formula only
2. Where will (does) baby usually sleep at night?	In parents’ bed Mattress/futon next to parents’ bed Cot next to parents’ bed Cot elsewhere in parents’ bedroom Cot in sibling’s room Cot in separate room
3. Will (does) baby ever sleep in bed with you?	No Yes^b^
4. Are you planning to use (currently using) routines each evening – such as bathing at a regular time – to help baby to settle at night?	No Yes Not thought about it
5. Do you think it is best to put your baby down to sleep at the same time each night, or when he/she is tired and starts to fall asleep?	Always settle when tired Almost always when tired Usually when tired Sometimes tired/sometimes at a regular time Usually same time Almost always same time Always same time
6. If baby wakes in the night, how many minutes are you likely to leave him/her to cry before you pick him/her up? (Record 0 min if you never leave him/her to cry)	______ Minutes
7. If baby wakes in the night, how many minutes will (does) it take before you feed him/her? (Record 0 min if you always feed immediately)	______ Minutes

a Newborn period wording asked for parents’ plans after they got home; wording at later ages (in brackets) asked about current practices.

b If yes, mothers answered two subsidiary questions:

(a) when baby sleeps in your bed with you at night, will this usually be (is this usually):

(i) all night (90–100% of the night); (ii) most but not all of the night; (iii) for short periods (eg, just for feeding); (iv) other (please specify) ________________.

(b) If you selected (i) or (ii) above, how many nights per week will this (does this) usually happen? (circle one): number of nights per week 1, 2, 3, 4, 5, 6, 7.

### Newborn assessments

Following earlier studies (Morrell, 1999; St James-Roberts *et al*., 2001; Tikotzky and Sadeh, 2009) the newborn ISFAQ was constructed to provide a brief screen for the mothers’ intended parenting strategies at home. Mothers answered seven questions by ticking boxes or inserting figures. Table 1 gives the wording of the ISFAQ items, including those used to assess response delay and feeding interval (items 6 and 7). Other items measured whether parents intended to use evening settling routines (item 4) and whether they planned to settle their baby to sleep at a regular time or when tired (‘settling method’, item 5).

### 2W assessments

Full written informed consent was obtained at a home visit when infants were 10–14 days old. Parents provided demographic information and the ISFAQ was repeated, re-worded to refer to current parenting practices. Researchers explained the Baby Day Diary (Diary) and asked parents to keep this for 3×24 h days. The Diary is a validated, real-time, parent-report measure of infant sleep, fuss/crying, and awake-settled behaviour (Barr *et al*., 1988; 2005).

### 5W assessments

ISFAQ current parenting and Diary measures were repeated. Following prior studies (Goodlin-Jones *et al*., 2001; Ball, 2007; Sitnick *et al*., 2008) researchers installed a self-focussing digital infra-red video camera (Sony HDR-XR200VE) on a tripod directed at the infant’s night-time sleep location, allowing up to 13 h of continuous recording. Parents were instructed in camera use and asked to switch it on when they began settling their infant to sleep at night and off the following morning. They were asked to follow their usual night-time habits. Parents decided when the camera was switched on at night and off the following morning and could switch the equipment off at other times if they chose to do so. The video was checked by researchers the following day and, if technical problems had arisen, one further attempt was made to obtain a recording.

### 3M assessments

The ISFAQ, Diary and video measures were repeated and parents completed the Brief Infant Sleep Questionnaire (BISQ, Sadeh, 2004). BISQ items include the average number of times/night parents detected infant waking, a widely used outcome measure of infant sleep-waking adopted here. Sleeping continuously for ⩾5 h/night is the criterion for settled night-time behaviour used in previous studies of this age-group (Moore and Ucko, 1957; Pinilla and Birch, 1993; St James-Roberts *et al*., 2001). To assess this, we asked: *In the last week, how many nights has your baby slept continuously for 5 hours or longer?* Answers were zero to seven nights. Because of extensive assistance with data collection, parents received high street shopping vouchers value £100 on returning the 3M data.

### Six-month (6M) follow-up

After a telephone follow-up, the ISFAQ, BISQ and 5-h sleep period measures were repeated and returned by mail.

### Data coding and analysis

The data were computer coded in Excel spreadsheets by researchers using written manuals and trained to ⩾90% reliability. Parental report data were coded blinded. Video coders cannot remain blinded, but video and parental report data were coded by different researchers. The Excel data were exported to SPSS 22 (IBM, 2013) for analysis.

The start time and end time of each diary behaviour period (sleeping, awake content, fussing or crying, feeding) was coded, allowing measures of the timing, duration and frequency of each behaviour. Following earlier findings, the diary night-time was defined as 7pm to 7am, day-time from 7am to 7pm (St James-Roberts *et al*., 2001). One night of video recording per infant at each age was coded. Video coding rules were based on Anders’ methods and conventional definitions of infant behaviour states (Anders and Keener, 1985; Goodlin-Jones *et al*., 2001). Detailed descriptions are in St James-Roberts *et al*. (2015). In summary, the videos were coded to identify five behaviour period types: awake; sleep; indeterminate; out of view; video turned off. Within each awake and sleep period, the times, frequencies and durations of infant behaviours (sleep, drowsy, awake content, fuss/crying, feeding), of ‘direct parental contact’ (touching, holding or speaking to an infant) and of ‘checking’ (approach to an infant without direct contact) were coded. To confirm reliability, 20 videos and 20 diaries were duplicate-coded by independent coders. Video between-coder Pearson correlations ranged from 0.862 to 1.00. Diary overall coding agreement was 0.998.

### Data analysis methods

Detailed ISFAQ, video and Diary data at each age within the first 3M are reported in a linked publication (St James-Roberts *et al*., 2016). In summary, compared with Bed-Sharing parents, General-Community parents were much more likely to delay responding to infant crying, to introduce an interval before feeding, and to settle infants for their night-time sleep at a regular time, at each age. These findings were replicated across methods and were the main parenting differences between these two groups. Building on these findings, analyses here examine whether each of these three elements of limit-setting versus infant-cued parenting predicts infant night-time sleep-waking at 3M and 6M.

Before examining the hypothesised predictions, parenting stability needed to be taken into account. One reason for this, reflecting Goodlin-Jones *et al*.’s (2001) findings, is that we expected the early postnatal weeks would be a transitional period for many parents as they tried out and adapted their methods of care. That is, some parents would not implement their planned forms of care, or would change them over time. Second, the limit-setting parenting literature implies that infants learn self-regulatory strategies which help them to sleep for long periods at night (Henderson *et al*., 2010; St James-Roberts *et al*., 2015). If so, consistent environmental cues may be important in supporting development of day: night differences in sleep-waking and extended night-time sleep periods. An inconsistent parenting environment might hamper this learning.

Following these rationales, data analysis involved three steps. First, employing a median-split method, the General-Community median newborn ISFAQ scores were used to divide parenting into infant-cued (above the median) versus limit-setting (below the median) categories for each ISFAQ measure (response delay, feeding interval, settling method) at each age. Table 2 shows the median values used. For response delay, this method allocated parents who reported responding to infant crying in 1 min or less to an infant-cued subgroup, and parents who took longer than 1 min to respond to a limit-setting subgroup, at each age. Similarly, parents who reported an interval before feeding of 1 min or less were allocated to an infant-cued feeding-interval subgroup, and parents who reported a pre-feed interval longer than 1 min were allocated to a limit-setting subgroup, at each age. For settling method, parents who reported settling when their infant was tired (always, almost always, usually or sometimes) were allocated to an infant-cued settling subgroup; parents who reported settling at a regular time (always, almost always, or usually) were assigned to a limit-setting subgroup. For each measure (response delay, feeding interval, settling method) we then cross-tabulated the numbers of parents in each group (General-Community; Bed-Sharing) who remained above the median, remained below the median, or who changed from above to below (or vice versa), between newborn and 5W ages. Potentially, this longitudinal analysis generated four parenting subgroups within each group (General-Community; Bed-Sharing) for each ISFAQ measure (response delay, feeding interval, settling method): stable infant-cued; increasingly infant-cued over age; increasingly limit-setting over age; stable limit-setting.

**Table 2:** Diary measures of feed length, feed interval and feed frequency at 2W and 5W in the groups and general-community subgroups

	Diary 2W measures						Diary 5W measures					
	Night-time feed length	Night-time feed interval	Night-time feed frequency	Day-time feed length	Day-time feed interval	Day-time feed frequency	Night-time feed length	Night-time feed interval	Night-time feed frequency	Day-time feed length	Day-time feed interval	Day-time feed frequency
Bed-Sharing group (*n*=19)^a^	0:24:00 (0:12:00)	1:37:00 (0:35:00)**	6.58 (2.20)**	0:19:00 (0:10:00)	1:13:00 (0:31:00)***	8.28 (2.78)***	0:18:00 (0:06:00)	1:47:00 (0:25:00)**	6.25 (1.49)***	0:17:00 (0:20:00)	1:21:00 (0:26:00)**	7.00 (3.26)**
GC group (*n*=101)^a^	0:23:00 (0:11:00)	2:08:00 (0:42:00)**	5.23 (1.97)**	0:23:00 (0:10:00)	1:48:00 (0:39:00)***	5.95 (2.06)***	0:22:00 (0:09:00)	2:29:00 (0:50:00)**	4.56 (1.77)***	0:20:00 (0:12:00)	1:57:00 (0:41:00)**	5.51 (2.13)**
GC response delay subgroups												
1. Stable ⩽1 min (*n*=35)	0:21:00 (0:11:00)	2:05:00 (0:39:00)	5.44 (1.70)	0:19:00 (0:08:00)*	1:41:00 (0:34:00)	6.36 (1.91)	0:17:00 (0:06:00)**	2:18:00 (0:41:00)	4.90 (1.61)	0:16:00 (0:06:00)**	1:48:00 (0:35:00)*	6.02 (2.07)
2. Changed from >1 to ⩽1 min (*n*=15)	0:26:00 (0:12:00)	1:50:00 (0:34:00)	5.82 (2.21)	0:26:00 (0:12:00)*	1:41:00 (0:43:00)	6.55 (2.88)	0:22:00 (0:11:00)**	2:14:00 (0:45:00)	4.99 (1.61)	0:20:00 (0:11:00)**	1:36:00 (0:30:00)*	6.65 (2.22)
3. Changed from ⩽1 to >1 min (*n*=20)	0:25:00 (0:12:00)	2:10:00 (0:45:00)	5.27 (2.13)	0:26:00 (0:11:00)*	1:55:00 (0:43:00)	5.58 (2.14)	0:25:00 (0:09:00)**	2:43:00 (1:03:00)	4.30 (2.88)	0:25:00 (0:10:00)**	2:11:00 (0:42:00)*	5.12 (1.82)
4. Stable >1 min (*n*=31)	0:24:00 (0:10:00)	2:15:00 (0:43:00)	4.98 (2.09)	0:25:00 (0:10:00)*	1:53:00 (0:42:00)	5.63 (1.57)	0:25:00 (0:09:00)**	2:34::00 (0:47:00)	4.30 (1.59)	0:23:00 (0:08:00)**	2:02:00 (0:43:00)*	5.55 (2.32)
GC feeding interval subgroups												
1. Stable ⩽1 min (*n*=20)	0:21:00 (0:14:00)	1:48:00 (0:33:00)*	6.08 (1.95)	0:21:00 (0:12:00)	1:32:00 (0:28:00)	6.82 (1.93)*	0:18:00 (0:06:00)	1:59:00 (0:32:00)**	5.39 (1.88)*	0:15:00 (0:06:00)*	1:38:00 (0:33:00)	6.48 (2.47)
2. Changed from >1 to ⩽1 min (*n*=14)	0:25:00 (0:12:00)	1:58:00 (0:45:00)*	5.69 (2.41)	0:24:00 (0:13:00)	1:36:00 (0:40:00)	6.86 (3.08)*	0:22:00 (0:13:00)	2:12:00 (0:35:00)**	5.11 (1.40)*	0:20:00 (0:12:00)*	1:50:00 (0:34:00)	6.03 (2.37)
3. Changed from ⩽1 to >1 min (*n*=36)	0:25.00 (0:11:00)	2:19:00 (0:46:00)*	4.89 (1.77)	0:24:00 (0:10:00)	1:58:00 (0:47:00)	5.54 (1.92)*	0:24:00 (0:09:00)	2:41:00 (0:54:00)**	4.18 (1.92)*	0:22:00 (0:08:00)*	2:07:00 (0:44:00)	5.36 (1.83)
4. Stable >1 min (*n*=31)	0:21:00 (0:09:00)	2:12:00 (0:36:00)*	5.08 (2.07)	0:24:00 (0:08:00)	1:52:00 (0:32:00)	5.51 (1.40)*	0:23:00 (0:09:00)	2:44:00 (0:47:00)**	4.09 (1.53)*	0:23:00 (0:09:00)*	2:01:00 (0:36:00)	5.42 (2.04)
GC settling method subgroups												
1. Stable ‘settled when tired’ (*n*=31)	0:25:00 (0:12:00)	2:01:00 (0:48:00)	5.71 (2.32)	0:24:00 (0:11:00)	1:38:00 (0:47:00)	6.52 (2.25)	0:21:00 (0:09:00)	2:10:00 (0:49:00)	5.37 (2.12)*	0:19:00 (0:09:00)	1:50:00 (0:49:00)	6.13 (2.67)
2. Changed from ‘settled at same time’ to ‘settled when tired’ (*n*=21)	0:22:00 (0:10:00)	2:10:00 (0:40:00)	5.22 (1.75)	0:23:00 (0:10:00)	1:51:00 (0:39:00)	5.76 (1.95)	0:22:00 (0:07:00)	2:29:00 (0:45:00)	4.40 (1.43)*	0:22:00 (0:10:00)	2:00:00 (0:34:00)	5.36 (1.51)
3. Changed from ‘settled when tired’ to ‘settled at same time’ (*n*=16)	0:23:00 (0:12:00)	2:04:00 (0:35:00)	5.44 (2.11)	0:23:00 (0:10:00)	1:50:00 (0:37:00)	5.89 (1.79)	0:21:00 (0:09:00)	2:40:00 (1:03:00)	4.28 (1.96)*	0:21:00 (0:08:00)	1:54:00 (0:38:00)	5.85 (2.00)
4. Stable ‘settled at same time’ (*n*=33)	0:24:00 (0:11:00)	2:14:00 (0:40:00)	4.86 (1.68)	0:23:00 (0:10:00)	1:54:00 (0:32:00)	5.71 (1.97)	0:23:00 (0:11:00)	2:40:00 (0:44:00)	4.08 (1.28)*	0:20:00 (0:10:00)	2:01:00 (0:40:00)	5.65 (2.03)

Data are mean (SD) frequencies or lengths of time in hours:minutes:seconds. ANOVA: *P<0.05; **P<0.01; ***P<0.001.

a Group and subgroup sizes given are at 2W. No 5W diary data were obtained from one Bed-Sharing and one General-Community case.

GC=General-Community.

In step two, objective, video or Diary, methods were used to confirm these group and ISFAQ-defined subgroup differences in parenting.

In step three, the parenting groups and subgroups were compared on video, diary and parental questionnaire measures of infant night waking and continuous sleeping at 3M and 6M of age.

## Results

### Parental questionnaire (ISFAQ) measures of parenting

Participants were predominately white, highly educated and married or co-habiting: Table 1 in St James-Roberts *et al*. (2016) provides detailed figures. The Bed-Sharing group contained fewer firstborn infants. Most General-Community infants (64%) but all Bed-Sharing infants were still exclusively breast milk fed at 3M. Most (93%) General-Community mothers planned their infants would usually sleep in cots in their bedroom, but 40–50% planned to or occasionally did bed-share with their baby for short periods (eg, feeding). All but one Bed-Sharing infant still bed shared through the night at 3M.

#### Response delay over age

The longitudinal stability analyses showed that Bed-Sharing parents’ reported responsiveness was highly stable, as well as rapid: 95% planning to and responding to infant cries within a minute at both newborn and 5W ages. Consequently, Bed-Sharing parenting could not in practice be divided into response delay subgroups.

In contrast, 34.3% of General-Community parents consistently responded to cries within a minute, 30.3% consistently delayed responding for >1 min, 15.2% reduced response delay, and 20.2% increased response delay, between newborn and 5W ages.

#### Feeding interval over age

Bed-Sharing parents were again highly stable and infant-cued in their reported parenting, so that 95% consistently reported feeding within a minute of detecting infant waking, making it impossible, in practice, to divide Bed-Sharing parents into feeding interval subgroups.

Among General-Community parents 20.2% consistently fed within 1 min, 29.8% consistently implemented a pre-feed interval >1 min; just 12.8% reduced the interval to ⩽1 min, and 37.2% increased the interval from ⩽1 to >1 min with age (the most common of the General-Community group’s four parenting subgroups).

#### Settling methods over age

Most (63%) Bed-Sharing parents planned and implemented settling their infants when tired (always, almost always, usually or sometimes). General-Community parenting was again less stable, so that 30.7% of these parents reported planning and implementing settling when tired, 32.7% consistently settling infants to sleep at a regular time, and 36.6% changed from one to the other (approximately equally in each direction).

These parent-report findings indicate that General-Community parenting was more unstable, as well as more limit-setting, compared with Bed-Sharing parenting. The General-Community group included stable and unstable parenting subgroups.

### Video or diary confirmation of the ISFAQ reports

#### Response delay

Table 3 summarises the 5W video data. In these recordings, Bed-Sharing infants signalled for a mean of 2 s before direct parental contact and 10 of 19 Bed-Sharing parents detected waking and intervened before infants signalled. On average, General-Community infants were awake for 3 min 32 s, and signalled for 1 min 3 s, before direct parental contact. These differences, examined in detail in St James-Roberts *et al*. (2016), are highly statistically significant.

**Table 3:** Video measures of night-time parenting behaviours at 5W in the groups and General-Community subgroups

	Video: infant behaviour when settled at bed-time						Video: parenting contact behaviours			
	% Infants asleep	% Infants drowsy	% Infants awake content	% Infants distressed	% Infants feeding	% Infants indeterminate	Mean (SD) interval between infant waking and direct parental contact	Mean (SD) interval between infant signalling and direct parental contact	Mean (SD) % of waking periods including direct parental contact	Mean (SD) % of sleeping periods including direct parental contact
Bed-Sharing group (*n*=18)^(c)^	50.0^(a)^	00.0^(a)^	11.1^(a)^	11.1^(a)^	22.2^(a)^	5.6^(a)^	0:00:14 (0:00:40)***	0:00:02 (0:00:08)**	89.03 (26.10)***	76.16 (00.06)***
GC group (*n*=99)^(c)^	49.5^(a)^	11.1^(a)^	24.2^(a)^	5.1^(a)^	00.0^(a)^	10.1^(a)^	0:03:32 (0:03:13)***	0:01:03 (0:01:33)**	26.70 (28.11)***	06.2 (0.21)***
GC response delay subgroups										
1. Stable ⩽1 min (*n*=34)	58.8	11.8	11.8	05.9	00.0	11.8	0:02:49 (0:03:03)	0:00:31 (0:00:54)*	32.2 (31.1)	10.1 (26.5)
2. Changed from >1 to ⩽1 min (*n*=15)	40.0	00.0	46.7	06.7	00.0	06.7	0:03:44 (0:03:22)	0:00:31 (0:00:26)*	25.0 (26.8)	00.6 (00.9)
3. Changed from ⩽1 to >1 min (*n*=20)	70.0	00.0	15.0	00.0	00.0	15.0	0:03:49 (0:03:39)	0:01:34 (0:02:27)*	23.5 (27.6)	07.7 (24.0)
4. Stable >1 min (*n*=30)	32.1	25.0	28.6	07.1	00.0	07.1	0:03:54 (0:02:58)	0:01:33 (0:01:31)*	22.8 (25.2)	03.9 (18.8)
GC feeding interval subgroups										
1. Stable ⩽1 min (*n*=20)	73.7	05.3	10.5	00.0	00.0	10.5	0:03:13 (0:03:01)	0:00:39 (0:01:09)	35.4 (35.3)	13.3 (31.2)
2. Changed from >1 to ⩽1 min (*n*=14)	33.3	00.0	50.0	08.3	00.0	08.3	0:04:17 (0:04:13)	0:01:02 (0:02:03)	35.1 (31.5)	07.0 (23.3)
3. Changed from ⩽1 to >1 min (*n*=36)	52.6	12.1	15.2	09.1	00.0	06.1	0:03:05 (0:03:07)	0:01:18 (0:02:04)	23.3 (25.8)	07.4 (24.5)
4. Stable >1 min (*n*=29)	32.1	17.9	28.6	03.6	00.0	17.9	0:04:09 (0:03:03)	0:01:01 (0:00:57)	19.5 (21.3)	00.6 (01.0)
GC settling method subgroups										
1. Stable ‘settled when tired’ (*n*=31)	58.1^(b)^	16.1^(b)^	19.4^(b)^	03.2^(b)^	00.00^(b)^	03.2^(b)^	0:02:45 (0:02:25)	0:00:34 (0:00:31)	22.2 (27.7)	09.5 (28.1)
2. Changed from ‘settled at same time’ to ‘settled when tired’ (*n*=20)	47.6^(b)^	09.5^(b)^	14.3^(b)^	09.5^(b)^	00.00^(b)^	19.0^(b)^	0:03:51 (0:05:05)	0:01:21 (0:01:28)	35.1 (34.1)	06.7 (20.8)
3. Changed from ‘settled when tired’ to ‘settled at same time’ (*n*=17)	56.3^(b)^	00.0^(b)^	12.5^(b)^	00.0^(b)^	00.0^(b)^	31.3^(b)^	0:02:51 (0:02:59)	0:01:30 (0:02:24)	26.5 (26.2)	03.2 (11.4)
4. Stable ’ settled at same time’ (*n*=31)	38.7^(b)^	12.9^(b)^	41.9^(b)^	06.5^(b)^	00.0^(b)^	00.0^(b)^	0:04:41 (0:04:01)	0:01:07 (0:01:44)	25.6 (24.9)	03.9 (17.9)

Data are percentages or lengths of time in hours:minutes:seconds. ANOVA *P<0.05; **P<0.01; ***P<0.001 ^(a)^Pearson *χ* ^2^ *P*<0.001; ^(b)^Pearson *χ* ^2^ *P*=0.013.

(c) No video data at 5W were obtained for two General-Community and one Bed-Sharing case.

3M=three months; 6M=six months; GC=General-Community; 5W=five weeks.

The General-Community subgroups of parents did not differ in how long they took to detect and respond to infant waking at 5W. However, the video-recorded difference in response delay to infant signalling at 5W was significantly different between these subgroups (Table 3). Tukey’s honest significant difference (HSD) tests allocated this difference between the four subgroups overall, but the data suggest a binary split: the two parent subgroups who reported delaying response at 5W being observed to let infants signal for a mean of around 1.5 min, compared with around 0.5 min for the two subgroups who reported responding within a minute (Table 3). These findings support the parents’ questionnaire reports and the median-split method.

#### Feeding interval

Consistently implemented intervals before feeding would be expected to lengthen the spacing between feeds. Because most General-Community infants were removed from the video recording for feeding at 5W (only nine of 99 were recorded feeding), diary data were used to measure these inter-feed intervals (the time between the end of one and start of the next feed). Table 2 presents the findings. General-Community infants had longer inter-feed intervals, and fewer feeds, than Bed-Sharing infants in the day and night at 2W and 5W.

The General-Community feeding interval subgroups differed in night-time, but not day-time, inter-feed intervals at both 2W and 5W (Table 2). Tukey’s HSD tests at 5W identified shorter night-time feed intervals in the subgroup who planned and implemented feeding within 1 min than in groups who planned and implemented, or implemented, pre-feed intervals >1 min (*P*<0.05). These subgroup differences in night-time inter-feed interval were substantial, averaging >20 min at 2W and >40 min at 5W (Table 2). These findings support the parents’ questionnaire reports and the median-split method.

#### Settling method

Settling at a scheduled time should reduce the number of infants already asleep or feeding when parents settled them for the night. As Table 3 shows, around half the infants in both groups were already asleep when video recording began. However, General-Community infants were more likely to be drowsy or awake content, and less likely to be feeding, when settled than Bed-Sharing infants.

Among the General-Community subgroups, infants who were consistently settled at a regular time appear more likely to have been awake content when settled than infants in the other subgroups (Table 3). Even so, nearly 40% of these infants were already asleep when settled for the night by parents.

These video findings provide some support for the parent-reported night-time settling method differences, but these group and subgroup differences were less robust than the differences in response delay and feeding interval.

#### Relationships between response delay, feeding interval and settling method

The concepts of infant-cued and limit-setting parenting imply a degree of overlap in parenting response delay, feeding interval and settling method. That is the case when the General-Community and Bed-Sharing groups are compared (Tables 2 and 3). Except for feed length, the groups differed substantially in all the Diary and video measures of all three parenting components.

In contrast, the General-Community response delay, feeding interval, and settling method subgroups were largely distinct in their video- and diary-measured parenting characteristics at 2W and 5W. An exception is that parents who planned and implemented rapid responding had shorter day-time feeds at 2W, and shorter day and night-time feeds and day-time feeding intervals at 5W (Table 2). Tukey’s HSD tests confirmed that this was mainly because the General-Community subgroup of parents who consistently responded rapidly also fed for shorter periods than both subgroups who delayed responding (*P*<0.05). This was not matched by significantly more frequent feeding, but both General-Community subgroups who responded rapidly at 5W tended to have short inter-feed intervals (Table 2). The General-Community subgroup who consistently settled infants when tired had more frequent night-time feeds at 5W than other subgroups (Table 2).

These overlaps in General-Community subgroup parenting are of a type consistent with the distinction between infant-cued and limit-setting parenting. However, the subgroup differences in Diary and video-measured parenting are much greater than their overlap, so that separate subgroup comparisons on outcome measures of infant sleep-waking are worthwhile.

### Hypothesis testing: comparing the parenting groups and subgroups on infant sleep-waking at 3M and 6M of age

Exact agreement between methods was not expected because of criterion and measurement differences. For instance, Diaries measured infants across three successive nights, defined as 7pm to 7am, while videos measured a single night defined by parents (around 9–10 h from 10pm). Nevertheless, group and subgroup differences should be similar across methods.

Bed-Sharing infants had more frequent, shorter sleep periods and woke more often during the night than General-Community infants on all video, Diary and questionnaire measures (Table 4).

**Table 4:** Comparison of the groups and General-Community subgroups on infant night-time sleep-waking behaviours at 3M and 6M of age

	3M			6M	
	Video: mean (SD) length of infant night-time sleep periods (hours:minutes:seconds)	Diary: mean (SD) length of infant night-time sleep periods (hours:minutes)	Questionnaire: mean (SD) no. of times infant woke per night	Questionnaire: mean (SD) no. of nights infants remained asleep for ⩾5 h periods	Questionnaire: mean (SD) no. of times infant woke per night
Bed-Sharing group (*n*=19)^a^	1:59:26 (1:00:40)**	2:04 (0:46)**	2.45 (0.78)**	2.94 (2.96)**	3.14 (2.21)***
GC group (*n*=101)^a^	3:33:28 (2:32:42)**	3:33 (1:56)**	1.59 (1.13)**	5.19 (2.45)**	1.30 (1.13)***
GC response delay subgroups					
1. ⩽1 min both newborn and 5W ages (*n*=34)	3:38:00 (2:49:59)	3:07 (1:27)*	1.90 (1.40)	4.41 (2.79)	1.56 (1.46)
2. Changed from >1 to ⩽1 min (*n*=15)	2:54:35 (1:16:17)	3:06 (1:11)*	1.33 (0.90)	5.57 (2.38)	1.25 (1.10)
3. Changed from ⩽1 to >1 min (*n*=21)	4:42:52 (3:21:41)	4:42 (2:58)*	1.27 (0.99)	5.83 (2.12)	0.92 (0.94)
4. >1 min both newborn and 5W ages (*n*=31)	3:03:22 (1:51:49)	3:24 (1:28)*	1.62 (0.97)	5.37 (2.20)	1.28 (0.80)
GC feeding interval subgroups					
1. ⩽1 min both newborn and 5W ages (*n*=20)	2:35:58 (1:37:57)**	2:41 (1:03)*	2.53 (1.45)***	3.83 (2.83)*	2.19 (1.44)**
2. Changed from >1 to ⩽1 min (*n*=14)	2:10:28 (1:13:47)**	2:54 (1:04)*	1.81 (1.29)***	4.73 (2.87)*	1.59 (1.16)**
3. Changed from ⩽1 to >1 min (*n*=37)	4:42:03 (3:24:24)**	4:07 (2:30)*	1.27 (0.99)***	5.93 (2.08)*	0.77 (0.97)**
4. >1 min both newborn and 5W ages (*n*=30)	3:30:17 (1:39:26)**	3:53 (1:55)*	1.20 (0.57)***	5.63 (1.92)*	1.13 (0.71)**
GC settling method subgroups					
1. ‘Settled when tired’ both newborn and 5W ages (*n*=31)	3:28:51 (2:09:58)	3:12 (1:13)	1.56 (1.05)	4.86 (2.63)*	1.66 (1.24)
2. Changed from ‘settled same time’ to ‘settled when tired’ (*n*=21)	3:34:16 (2:37:44)	3:10 (1:45)	1.93 (1.37)	4.10 (2.69)*	1.45 (1.00)
3. Changed from ‘settled when tired’ to ‘settled at same time’ (*n*=16)	3:42:45 (3:17:12)	3:42 (2:14)	1.66 (1.27)	5.50 (2.34)*	1.00 (0.76)
4. ‘Settled at same time’ both newborn and 5W ages (*n*=33)	3:32:48 (2:32:18)	4:02 (2:22)	1.37 (0.96)	6.06 (1.88)*	1.00 (0.91)

Data are numbers or lengths of time in hours:minutes:seconds. ANOVA: *P<0.05; **P<0.01; ***P<0.001.

a Group and subgroup sizes given are at 3M. At 6M, eight General-Community and one Bed-Sharing case did not return data.

3M=three months; 6M=six months; GC=General-Community; 5W=five weeks.

Among the General-Community subgroups, delayed responding predicted longer night-time sleep periods in 3M Diary, but not in video or parental questionnaire measures, or in 6M measures (Table 4), providing weak support for our primary hypothesis.

Our secondary hypothesis predicted that use of feeding intervals in the early weeks would lead to longer day-time feeds at 5W to compensate for the longer feeding intervals and fewer feeds at night. As Table 3 shows, the figures support that expectation. At 5W, each day-time feed of the subgroup who consistently implemented a pre-feeding interval lasted 8 min longer, on average, than the feeds of the subgroup who consistently minimised night-time pre-feeding intervals (Tukey’s HSD test difference *P*<0.05).

**Figure 1:**
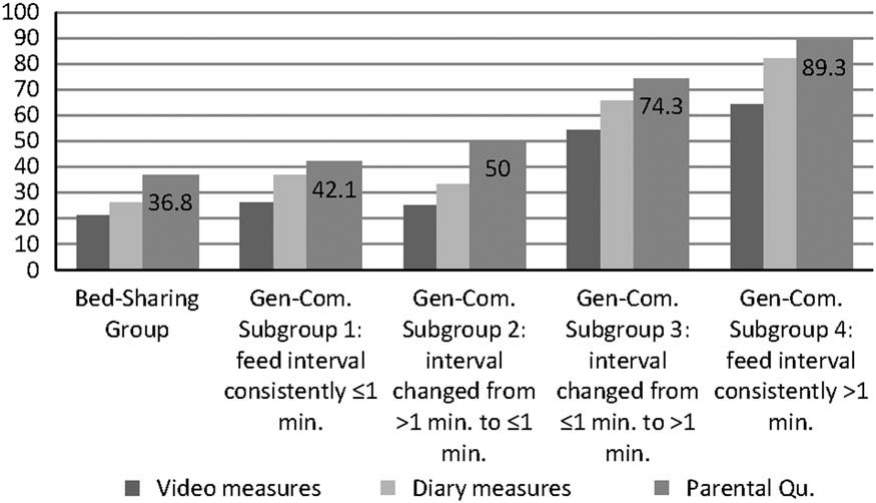
Percentages of infants in the Bed-Sharing group and each General-Community feed interval subgroup who remained asleep for ⩾5 h periods at night at three months of age: video, diary and questionnaire measures

Stably implemented feeding intervals also predicted the infant outcome sleep-waking measures from all three methods at 3M, and both questionnaire measures at 6M age (Table 4). Figure 1 shows the number of infants in the Bed-Sharing group, and General-Community feeding interval subgroups, who remained asleep for periods of ⩾5 h at night at 3M measured by video, Diary and questionnaire methods. Like Bed-Sharing infants, only ~40% of General-Community infants who were fed within a minute at both newborn and 5W ages were asleep for continuous night-time periods of 5 h or more at 3M. In comparison, infants whose parents planned and implemented an interval before feeding were around twice as likely to remain asleep for ⩾5 h night-time periods, at 3M.

The parents’ bed-time settling method did not predict 3M infant sleep-waking on any measure (Table 4). The prediction of questionnaire-reported sleep periods lasting ⩾5 h at 6M may be a chance finding but, in any case, settling method did not predict infant sleep-waking robustly.

Stepwise logistic regressions were used to examine whether the feeding interval predictions in Figure 1 were affected by the response delay scores (see Table 5). The feeding interval predictions all remained highly significant and were mostly unaffected by adding or removing the response delay scores. The exception was that adding response delay improved the prediction of Diary-measured sleep periods lasting 5 h or more at 3M. However, the effect size was small and, when entered in the first step, the response delay scores alone failed to predict any of the measures of 3M infant sleeping (Table 5).

**Table 5:** Results of stepwise logistic regressions using feed interval to predict the video, Diary and questionnaire measures of the infant ⩾5 h sleep periods at 3M included in Figure 1^a^

	*χ* ^2^ for step	*P* for step	Nagelkerke *R* ^2^ for model
Video measures			
Feed interval	14.1	<0.001	0.20
Response delay	0.12	0.73	0.20
Diary measures			
Feed interval	9.05	0.003	0.12
Response delay	5.02	0.025	0.19
Questionnaire measures			
Feed interval	13.0	<0.001	0.18
Response delay	3.08	0.08	0.21

a Feed interval was entered in the first step and response delay added in the second step.

The *χ* ^2^ figure gives the goodness of fit for the step; the Nagelkerke *R* ^2^ estimates the effect size. Entering response delay in the first step did not predict the infant sleep measures significantly.

#### Possible confounders

First and later-born infants did not differ in 3M or 6M sleep-waking, while the consistency of the evidence across methods, groups and subgroups rules out the group difference in video indeterminate time, described in St James-Roberts *et al*. (2016), as an explanation of the findings. Analyses reported in St James-Roberts *et al*. (2015) found that 66% of infants exclusively fed breast milk, and 62.5% of infants fed formula or mixed breast and formula milk at 3M, were settled for ⩾5 h night-time periods at 3M, which is not significantly different.

## Discussion

There is evidence that RCTs of parenting programmes can be confounded by parents’ unwillingness to implement interventions that conflict with their values or circumstances [Medical Research Council (MRC), 2007; Olds *et al*., 2007]. Accordingly, following MRC (2007) guidelines, this study was designed to complement existing RCT evidence by using video recording and other methods to observe London infants’ and parents’ typical night-time behaviours in their normal home environments. Assessments focussed on the first 3M of age: the period when most western infants develop prolonged sleep periods at night. A General-Community group of 101 infants and parents, most of whom were expected to adopt limit-setting parenting methods, was compared with a group of 19 planned Bed-Sharing parents and babies, who were expected to adopt highly infant-cued parenting methods. This comparison group included parents who intended to bed-share from before their baby’s birth and did so consistently, unlike ‘reactive’ bed-sharers who respond to their infant’s night waking and signalling by switching to bed sharing (Germo *et al*., 2007).

The first finding was that around a third of London General-Community parents did not implement their planned form of parenting, or changed from an infant-cued towards a limit-setting strategy, or vice versa, in the first five postnatal weeks. This variability, initially described by Goodlin-Jones *et al*. (2001), stood in marked contrast to the stability in infant-cued parenting shown by the Bed-Sharing group. These parenting inconsistencies are important in their own right. For instance, some parents may manage the infant crying peak at one to two months of age (Barr, 1990) by increasing responsiveness, so that parenting is reactive in such cases. The implication is that research is needed to understand why parents change care strategies and whether anticipatory guidance can help to prevent unintended consequences, such as reactive bed sharing. This finding also has implications for RCT design. Even recent RCTs of parenting programmes for preventing infant sleep problems have provided little evidence that parents consistently implement the intended interventions or differ from control groups (Stremler *et al*., 2013; Hiscock *et al*., 2014). RCTs need to document implementation and reasons for implantation failure.

The second finding was that stable limit-setting parenting led to longer infant night-time sleep periods at 3M and 6M of age. Analyses of the videos confirm that this involved increased infant sleep length over age, not variations in parents’ detection of infant waking (St James-Roberts *et al*., 2015). Supporting our secondary, but not primary, hypothesis one parenting strategy – consistently implementing a short interval before feeding – led to fewer night-time feeds, longer night-time feed intervals, and longer day-time feeds at 5W, and twice as many infants sleeping continuously for 5 h or more at night at 3M, compared with immediate or rapid feeding. A proviso is that 11% of infants were not asleep for ⩾5 h periods at night at 3M even where parents consistently implemented an interval before feeding. This is in keeping with transactional models which posit that infant, as well as parenting, factors influence infant sleep-waking development (Sadeh *et al*., 2010). The nature of these infant factors requires further research.

These findings need to be interpreted in light of the methodological limitations of this study, including that participants were highly educated, largely white parents in stable relationships in London, the small size of the comparison group and General-Community subgroups, and reliance on only three 24-h diary days at each of three ages and one night of video recording at each of two ages. The use of median splits to divide the General-Community group into subgroups at each age has disadvantages, but these concern the likelihood that analyses will fail to identify subgroup differences. In contrast, our video and diary findings confirmed the validity of our median-split analyses, while the finding of highly significant differences in the group and General-Community subgroup measures of infant sleep-walking across video, diary and questionnaire methods at 3M of age, and at both 3M and 6M ages, is highly unlikely to be due to error or chance.

As others point out (Tikotzky *et al*., 2015) a crucial limitation of the longitudinal observation method is that it cannot prove causal relationships between study variables. It follows that factors other than feeding interval, including infant characteristics, may be responsible for the infant outcomes observed. Although cross-lagged and other statistical methods could be used to explore causality, they too have limited power to resolve this question.

Although we accept this limitation, four RCTs – the most powerful research method for establishing causation – have provided replicated evidence that limit-setting parenting increases the proportion of infants who have long night-time sleep periods by 3M of age (Wolfson *et al*., 1992; Pinilla and Birch, 1993; St James-Roberts *et al*., 2001; Symon *et al*., 2005). Mirroring findings here, one RCT found that parents did implement feeding intervals (St James-Roberts *et al*., 2001) and another that limit-setting parenting, including feeding intervals, reduced night-time feed frequency and increased day-time feeds at four to six weeks, and night-time sleep periods at 3M of age (Pinilla and Birch, 1993). The current study’s value lies in complementing these RCT findings by adding evidence that these parenting features are associated with the development of prolonged infant night-time sleeping in representative home environments during the period when most western infants become settled at night. Our findings are also consistent with observational evidence that infants in general increase their day-time feeds at around two months of age (Wright, 1993) and that infants whose mothers nurse them less at bedtime show a steeper increase in night-time sleep (Philbrook and Teti, 2016). In addition, they complement RCT evidence that limit-setting interventions are particularly effective where infants feed frequently (Nikolopoulou and St James-Roberts, 2003; Hiscock *et al*., 2014). In this study, increasing the interval before feeding as infants aged was the General-Community parents’ most common longitudinal parenting strategy, suggesting that parents who implemented this from birth adopted a form of parenting which is normative as infants grow older. Evidence that parenting typically becomes less infant-cued as infants age has been found, too, in other English (Williams *et al*., 2016) and Norwegian (Sudnes and Andenaes, 2016) studies. It seems likely that parents’ initial concern about their baby’s well-being and weight gain gives way to a more deliberated approach as infants develop.

Taken together, these findings provide substantial, but not conclusive, evidence that a short interval before feeding leads to a cascade of adaptations in infant self-regulation and sleep-waking which many infants manage successfully by 3M of age. RCT confirmation of this finding is needed, but physiological research to establish how feed spacing can alter infant metabolic self-regulation may be a useful prior step. For instance, an interval before feeding may increase wakefulness and feeding vigour, resulting in greater intake and physiological adjustments which extend sleep length and the interval before the next feed. The existing findings do not support the long-standing assumption that breast milk constituents require 3M-old infants to wake frequently at night.

For clinical purposes, any benefits which stem from introducing an interval before feeding need to be balanced against its disadvantages, including the evidence that this leads to increased distress in General-Community infants, relative to infants whose parents use highly infant-cued parenting including bed sharing. However, no significant differences in infant night-time distress were found between General-Community subgroups with minimal versus typical pre-feeding intervals (St James-Roberts *et al*., 2016). Moreover, the difference in distress between General-Community and Bed-Sharing groups was larger at 2W (~30 min per night) than at five or 12 weeks of age (~12–13 min per night). While some parents may consider that any infant distress should be avoided, others may judge that a night-time increase of around 1 min/h after 5W of age is justified by the potential benefits of implementing a short interval before feeding.

These findings can be conveyed to parents and professionals to help them to make informed choices but, in our view, should not be incorporated prescriptively into health service recommendations. Instead, the findings should provide the basis for further research to substantiate, and refine, their use. The existing evidence implies that limit-setting parenting should result in less infant night-time distress and improved sleeping over the long-term, while improved sleeping should help to support healthy infant development (El-Sheikh and Sadeh 2015). However, there is little direct evidence to confirm these overall benefits, or those of infant-cued parenting.

‘Authoritative’ parenting, that combines warmth with limit-setting, is especially effective in supporting older children’s development (Pratt *et al*., 1988). Perhaps because of contemporary concerns with ‘baby-friendly’ parenting and maintaining breast-feeding, the question of how and when parenting can support infant sleep-waking and other aspects of development has received sparse attention. Instead of perpetuating the debate about the ‘best’ form of parenting, the question of *when* to transition from infant-cued to limit-setting parenting so as to maintain breast-feeding, support infant self-regulation, and prevent long-term night waking and distress, seems likely to be a fruitful focus for research.
